# Copy number variants encompassing Mendelian disease genes in a large multigenerational family segregating bipolar disorder

**DOI:** 10.1186/s12863-015-0184-1

**Published:** 2015-03-15

**Authors:** Rachel L Kember, Benjamin Georgi, Joan E Bailey-Wilson, Dwight Stambolian, Steven M Paul, Maja Bućan

**Affiliations:** Department of Genetics, University of Pennsylvania, Philadelphia, PA USA; Department of Ophthalmology, University of Pennsylvania, Philadelphia, PA USA; Department of Psychiatry, Perelman School of Medicine, University of Pennsylvania, Philadelphia, PA USA; Appel Alzheimer’s Disease Research Institute, Mind and Brain Institute, Weill Cornell Medical College, New York, NY USA; Computational and Statistical Genomics Branch, National Human Genome Research Institute, National Institutes of Health, Baltimore, MD USA

**Keywords:** CNV, Bipolar disorder, Family based studies, Mendelian disease genes, Genetics loci

## Abstract

**Background:**

Bipolar affective disorder (BP) is a common, highly heritable psychiatric disorder characterized by periods of depression and mania. Using dense SNP genotype data, we characterized CNVs in 388 members of an Old Order Amish Pedigree with bipolar disorder. We identified CNV regions arising from common ancestral mutations by utilizing the pedigree information. By combining this analysis with whole genome sequence data in the same individuals, we also explored the role of compound heterozygosity.

**Results:**

Here we describe 541 inherited CNV regions, of which 268 are rare in a control population of European origin but present in a large number of Amish individuals. In addition, we highlight a set of CNVs found at higher frequencies in BP individuals, and within genes known to play a role in human development and disease. As in prior reports, we find no evidence for an increased burden of CNVs in BP individuals, but we report a trend towards a higher burden of CNVs in known Mendelian disease loci in bipolar individuals (BPI and BPII, p = 0.06).

**Conclusions:**

We conclude that CNVs may be contributing factors in the phenotypic presentation of mood disorders and co-morbid medical conditions in this family. These results reinforce the hypothesis of a complex genetic architecture underlying BP disorder, and suggest that the role of CNVs should continue to be investigated in BP data sets.

**Electronic supplementary material:**

The online version of this article (doi:10.1186/s12863-015-0184-1) contains supplementary material, which is available to authorized users.

## Background

Bipolar affective disorder (BP) is a serious mental disorder characterized by periodic changes in mood, energy and activity levels alternating between episodes of depression and mania [1]. The lifetime prevalence of BP type I (BPI) and type II (BPII) is 2.1% in the United States [2] and the age of onset is early, at 18–19.5 years old [3], making BP responsible for the loss of more disability-adjusted life-years than all forms of cancer [4] and consequently it is a major public health concern [5]. As with many complex disorders, the underlying etiology of BP is unknown, but is hypothesized to be the result of multiple gene-gene and gene-environment interactions [6]. Epidemiological studies using twin data show that BP has heritability estimates ranging from 62-89% [7,8], although the mode of inheritance is complex. Common genetic factors have been shown to contribute substantially to susceptibility for bipolar disorder, with a strong polygenic contribution [9]. Several potential BP candidate genes have been described [10], but findings are inconsistent and the role of specific genes in BP is currently undetermined.

Copy number polymorphisms (CNVs) are a common class of genetic variation in the human genome [11-14], and can be readily detected using intensity data from genome-wide SNP arrays. Like single-nucleotide polymorphisms (SNPs), CNVs can affect gene expression, either by encompassing genes or regulatory elements. Large, cytogenetically detectable chromosomal rearrangements, such as aneuploidy, have been historically linked to human disease [15]. Studies of several genomic disorders, associated with inherited or sporadic genomic anomalies which are smaller (<5 MB) and therefore can not be detected using conventional cytogenetic methods, revealed that deletions and duplications encompassing several genes may lead to complex and highly pleiotropic clinical syndromes [16].

Several systematic surveys of copy number variation using Comparative Genomic Hybridization (CGH) and high-density SNP arrays revealed a large number of benign deletions and duplications across the genome, but also revealed the role of a large number of rare potentially pathogenic CNVs in neurodevelopmental and psychiatric diseases, particularly in Autism Spectrum Disorder and Schizophrenia [17]. In Autism Spectrum Disorder, CNVs were found in a number of chromosomal regions [18], and the burden of rare and *de novo* CNVs was enriched in affected individuals compared to controls and their unaffected siblings [19]. Similarly, several large, rare CNVs have been associated with schizophrenia [20,21]. Among these CNVs several have been observed at elevated rates in multiple neurodevelopmental and psychiatric disorders [22,23].

Both linkage and candidate gene analyses, as well as genome-wide association studies, indicate a shared genetic architecture and an overlap of susceptibility between BP and schizophrenia [24]. However, compared to studies conducted on ASD and schizophrenia, there are far fewer examples of CNVs associated with BP [25]. An analysis of 1001 cases and 1034 controls reported an increased burden of singleton CNVs in early onset bipolar cases [26]. Also, in an independent study of 788 trios, frequencies of *de novo* CNVs were significantly higher in bipolar disorder as compared to controls, but not as high as in schizophrenia [27]. However, a study using Welcome Trust Case Control Consortium (WTCCC) data found no evidence for an elevated burden of CNVs in bipolar individuals (n = 1697) compared to controls (n = 2806), although the burden was found to be elevated in schizophrenia [28]. The same authors recently published the most comprehensive analysis of CNVs in the WTCCC revealing a significantly lower rate of rare very large CNVs (>1 Mb) in patients with bipolar disorder (n = 1,650) compared to reference individuals without psychiatric disorder (n = 10,259) [29]. Although the authors state that this result needs to be verified in larger datasets, they propose that a lower CNV burden may underlie differences in the presentation of clinical phenotype between bipolar disorder and schizophrenia. In addition, recent research suggests that *de novo* CNVs may play a smaller role in BP compared to schizophrenia [30], but the role of inherited CNVs remains uncertain.

The Old Order Amish are a founder population originating in middle Europe. Since 1964, when Victor McKusic and colleagues described the benefits from medical genetics studies in the Amish [31], a large number of Mendelian disorders have been described in this population [32]. More recently, next generation sequencing studies of neurodevelopmental and psychiatric disorders in the Amish provide a unique opportunity to address the role of rarer forms of genetic variation [33,34]. However, these recent studies focus on the role of single nucleotide variants (SNVs). Apart from a handful of gene deletions associated with Mendelian disease [32], and 50 CNV regions identified in a subset of individuals from the Old Order Amish pedigree with bipolar disorder [35], global analysis of copy number variation has not been systematically carried out in this genetic isolate.

The aim of the present study was to investigate CNVs in the extended Old Order Amish pedigree with bipolar disorder, and compare these CNVs with CNVs detected in a large collection of unrelated control subjects to identify deletions and duplications private to this family. Also, we compared burden and frequency of CNVs in family members with affective disorders (BPI, BPII and MDD-R) with their unaffected relatives to identify CNVs potentially contributing to the locus and allele heterogeneity of bipolar disorder. Our systematic analysis revealed 67 rare and moderately rare CNVs encompassing Mendelian disease genes that may contribute to the complex and pleiotropic manifestation of mental illness in this founder population.

## Results

### Overall strategy

**Figure 1:**
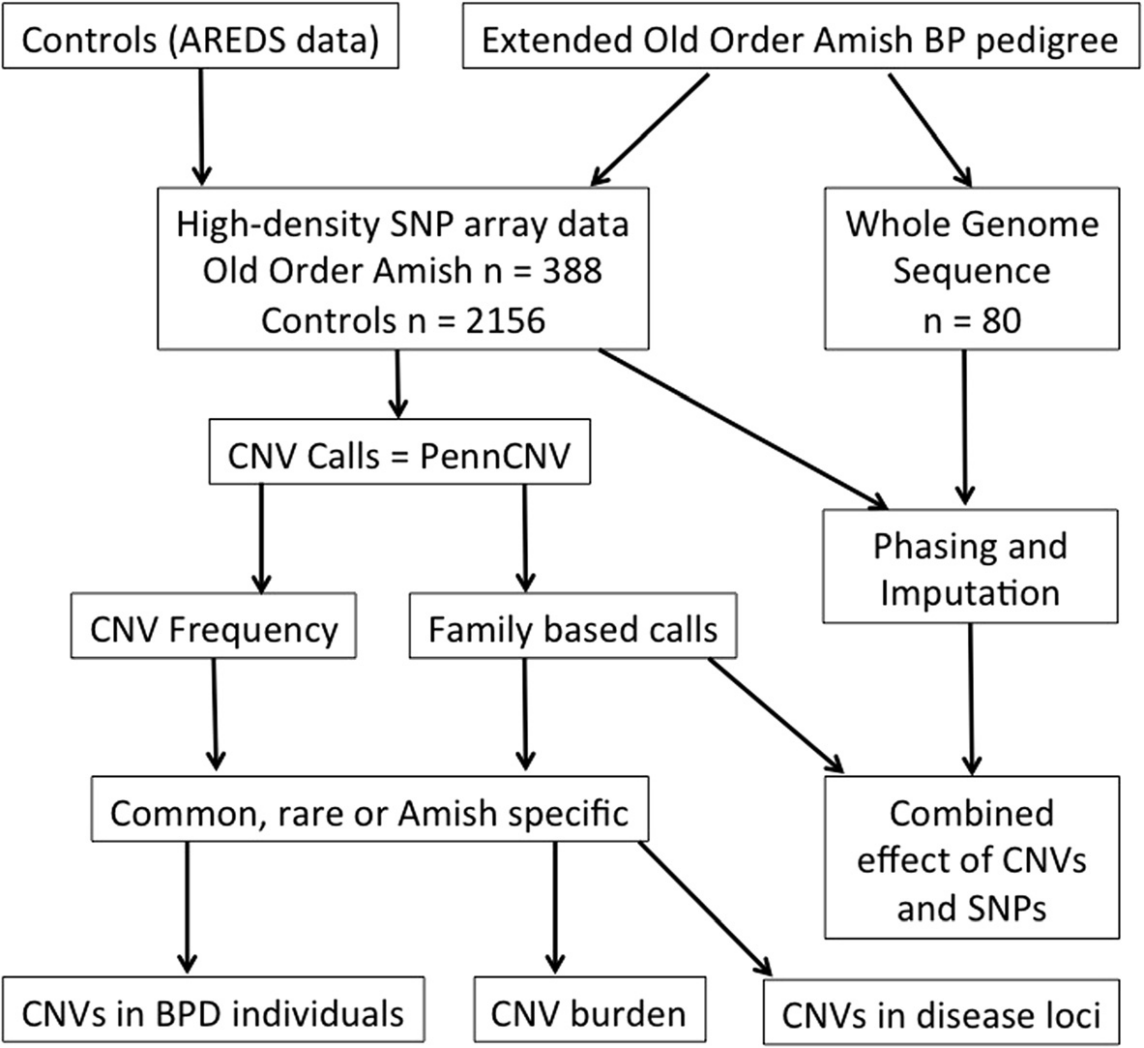
**Flowchart outlining the quality control and analysis pipeline of this study.** SNP data from the control and Old Order Amish populations was used to call CNVs using the PennCNV program. The pedigree structure in the Amish allowed family based calls to be made, and the CNV frequency in the control data allowed us to determine whether the Amish CNV calls were common, rare, or exclusive to the Amish population. We sought to determine CNVs in bipolar individuals, burden in individuals, and CNVs in disease loci. In addition, we utilized the whole genome sequence data to find CNVs and SNPs within the same gene in the same individual.

To characterize structural variants in 388 members of a large multigenerational Old Order Amish pedigree with bipolar disorder, we used dense SNP genotype data generated using the Illumina Omni 2.5 M platform [33]. We also performed CNV analysis on 2,156 Age-related Eye Disease Study (AREDS) control subjects (1,897 with European ethnicity) genotyped using the same SNP platform. A flowchart (Figure 1) outlines the quality control and analysis pipeline employed to address: a) differences in the allele frequency of CNVs in this genetic isolate compared to a large sample of subjects of European origin; b) the role of CNVs in susceptibility to bipolar illness in this large pedigree; c) an estimate of the total per genome (or person) burden of CNVs, including CNVs that encompass known disease loci.

### A catalog of inherited CNVs in an Amish pedigree segregating bipolar disorder

As part of our genetic study of bipolar disorder in the Old Order Amish, we analyzed genome-wide SNP genotype data using the Penn CNV algorithm [36] to identify CNVs. We examined the breakpoints of all CNVs (n = 18,986) and clustered groups of CNVs that have arisen from common ancestral mutations (see Methods) into CNV regions (n = 561). Using the pedigree relationships, we classified all regions as either ‘inherited’ (shown to pass from parent to child), or of ‘unknown origin’ (not seen in either parent). To avoid possible technical artifacts in the analysis, we focused on the high quality, inherited CNVs observed in multiple (more than three) Amish family members. These variants are less likely to represent false positives in computational prediction or rearrangements that arose during culturing of lymphoblastoid cell lines [37]. Furthermore, in a large pedigree with an excess of bipolar disorder, we expect that the causal genetic variants will be inherited, rather than *de novo*. Of 541 inherited CNV regions identified by the analysis of 328 family members (a subset that contains both parent and child information), 33 overlap with 50 regions previously identified in a small scale study of the core family, i.e. 50 family members [35]. Eight CNV regions (four which are exonic, one which is intronic, and three which are intergenic) were detected on Chromosome X (Additional file 1: Table S2).

**Figure 2:**
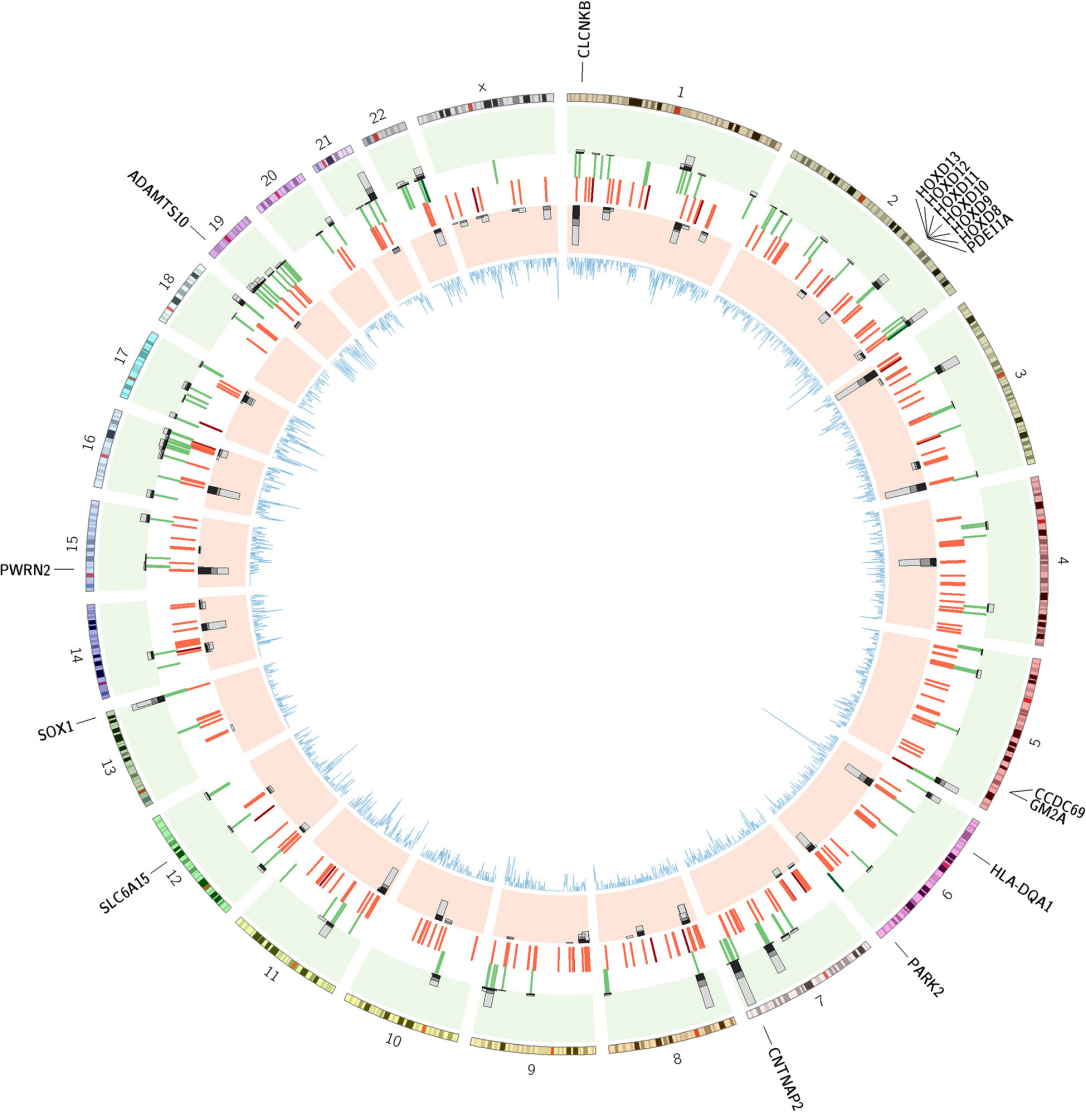
**Location of CNV Regions,** **burden of rare CNVs,** **and disease genes.** CNV regions are shown as red and green lines (green: heterozygous duplication, dark green: homozygous duplication, red: heterozygous deletion, dark red: homozygous deletion). Stacked histogram bars represent the location of specific rare CNVs, and the number, split by phenotype (green background: duplications, red background: deletions; dark grey: bipolar, mid-grey: unknown, light grey: unaffected). Inner line plot (blue) shows location and number of disease genes. Genes of interest are labeled around the outside of the plot.

Among detected inherited CNV regions, the largest category consisted of common CNVs (present in more than 5% of controls) found throughout the large multigenerational Amish pedigree. In addition we detected 104 moderately rare (present in less than 5% of controls) and 139 rare (present in less than 1% of controls) CNV regions in subjects, as well as 129 ‘exclusive’ regions that were not present in any controls. Of these exclusive regions, 36 are deletions and 93 are duplications, and 99 regions include genes (Figure 2, created using Circos [38]). These ‘exclusive’ variants form a key part of the genomic architecture of this pedigree, and could play a role in phenotypic presentation. To illustrate the frequency of a CNV in the pedigree, CNV counts are presented for nuclear families only in which the CNV is present, and only for those individuals with bipolar or well phenotypes; individuals with unknown or other phenotypes are excluded. They include a 26 kb duplication on 13q24, present in 109 Amish individuals (affected 23/86, 26.4%; unaffected 66/232, 28.4%), which encompasses the entire SRY (sex determining region Y)-box 1 (*SOX1*) gene; a 24 kb deletion on 5q33.1, found in 48 individuals (affected 8/38, 21.1%; unaffected 29/79, 36.7%), in an intergenic region upstream of both coiled-coil domain containing 69 (*CCDC69*) and GM2 ganglioside activator (*GM2A*); and a 10 kb deletion on 12q21.31, found in 33 individuals (affected 9/25, 36.0%; unaffected 19/51, 37.3%), located downstream of solute carrier family 6, member 15 (*SLC6A15*).

We compared the total number, average size, and burden per individual of CNVs in a) all family members (n = 375), b) subjects with bipolar disorder (n = 77), and unaffected Amish and control individuals (Amish n = 234, controls n = 1897) (Table 1). Analysis was performed on copy number losses (deletions) and gains (duplications) separately. Overall number of detected deletions and duplications in the Amish pedigree were comparable to those detected in the control subjects.

**Table 1:** **Summary of CNV calls for the Old Order Amish** (**n** = **375**) **and controls** (**n** = **1897**)

		**Total**			**Deletions**			**Duplications**		
		**Amish CNVs**	**Amish Inherited CNVs**	**Controls**	**Amish CNVs**	**Amish inherited CNVs**	**Controls**	**Amish CNVs**	**Amish inherited CNVs**	**Controls**
Total Number	All Samples	18986	6345	77205	10942	4154	51138	8044	2191	26067
	Affected	3844	1380	-	2216	905	-	1628	475	-
	Unaffected	12191	3962	-	6922	2581	-	5269	1381	-
Average Size (bp)	All Samples	36123	42064	29697	31124	32701	23768	42924	59815	41328
	Affected	41123	44572	-	39802	33575	-	42921	65523	-
	Unaffected	34363	39932	-	29072	32093	-	41315	54581	-
Burden (per individual)	All Samples	50.6	23.2	40.7	29.2	15.2	27.0	21.6	8.2	13.7
	Affected	49.9	23.4	-	28.8	15.3	-	21.1	8.1	-
	Unaffected	52.1	23.7	-	29.6	15.5	-	22.5	8.3	-

Total number, average size, and burden of CNVs was calculated for all Amish CNVs, inherited Amish CNVs, and control CNVs. CNVs were analyzed together, and as deletion and duplication events separately.

### CNVs and disease association

No individual CNV segregated fully with bipolar disorder. Analysis of CNV data in the linkage regions previously reported [33] identified a single duplication event in the 7q21 region. The 95 kb duplication localizes upstream of the maximum LOD score marker *D7S518*. We confirmed inheritance of the CNV on the 4-4-1-4 haplotype (*D7S2431*-*D7S554*-*D7S518*-*D7S2509*). The duplication spans the first exon of the collagen, type XXVI, alpha 1 (*COL26A1*) gene. *COL26A1* has yet to be functionally characterized, with a possible role in aspirin-intolerant asthma [39].

Burden analysis of CNV regions in genes in the Amish shows a trend towards an increased number of these CNVs in bipolar individuals (narrow phenotype: BPI and BPII), although this does not reach significance (narrow burden: 17.3, unaffected burden: 15.6, p = 0.11) (Table 2). We identified three rare deletions in *KCNJ6*, *UNC13C*, *OTOL1* and 7 rare duplications in *CNTNAP2*/*MIR548F3*, *CORO7*/*VASN*, *DTNB*, *EMID2*, *KCNF1*, *PDPR and SGTA*/*THOP1* that are present in children with bipolar disorder (and their parents). In addition, we find other rare CNVs in genes that are present frequently in individuals with bipolar disorder (Table 3). Association analysis for all CNV regions was performed using two different methods: a) FBAT [40] and b) EMMAX [41], although no CNV was found to be significantly associated with BP following correction for multiple testing. We found no overall enrichment of large inherited CNVs in affected individuals, although 7 large, rare, CNVs in genes occurred more frequently in subjects with bipolar disorder than unaffected family members and control subjects. One of the largest rare genic CNVs is the previously reported 150 kb deletion in the 15q11 region, which encompasses the entire Prader-Willi region non-protein coding RNA 2 (*PWRN2*) gene [35]. The deletion is present in 15 families, is found on two haplotypes (*D15S817*-*D15S1021*-*D15S128*: 3-6-3 and 3-5-3) and is widely spread throughout the pedigree; 20/32 (62.5%) of those with bipolar disorder in these carrier families have the CNV, compared to 28/52 (53.8%) of well individuals.

**Table 2:** **Burden analysis of CNV Regions reveals a higher number of CNVs in genes in individuals with narrow bipolar phenotype** (**BPI and BPII**)

	**Unaffected**	**Broad**	**Narrow**
No. All CNVs	28.1	28.7	29.5
P vs unaffected	-	0.39	0.26
No. All CNVs in genes	15.6	16.8	17.3
P vs unaffected	-	0.16	0.11
No. Rare CNVs in genes	9.7	11.0	11.3
P vs unaffected	-	0.12	0.09
No. CNVs in disease genes	4.8	5.5	5.7
P vs unaffected	-	0.11	0.06
No. Rare CNVs in disease genes	4.0	4.8	5.0
P vs unaffected	-	0.07	0.06

A trend towards an increased number of CNVs in disease genes in individuals with narrow bipolar phenotype is also reported.

**Table 3:** **CNVs within genes**

**Cytoband**	**Start**	**Stop**	**No. snp**	**length** **(bp)**	**CN**	**Contained genes**	**Frequency**	**No. subjects**	**No. affected**	**No. families**	**Predicted effect**	**FBAT**	**EMMAX**	**Previous disease associations for gene**
1p36.21	13171723	13218942	31	47220	0&1	*LOC440563*	Rare	38	13	15	Gene del	1	0.1887	
1q22	155152205	155162067	22	13287	3	***MUC1***, *TRIM46*	Amish specific	28	13	32	Partial gene dup	0.0711	0.1535	Kidney disease [42]
1q24.1	165644865	165649715	9	4851	1	*ALDH9A1*	Very rare	17	7	12	Exonic del	1	0.1434	
2q31.1	176929113	177000696	86	71584	3	*EVX2*, ***HOXD10***, *HOXD11*, *HOXD12*, ***HOXD13***, *HOXD8*, *HOXD9*	Amish specific	45	15	32	Gene dup	0.4122	0.9834	Limb and genital abnormalities [43,44]
2q37.3	241500674	241516094	20	24920	3	*DUSP28*, *RNPEPL1*	Very rare	74	13	43	Partial gene dup	0.4226	0.3229	
2q37.3	241482099	241516094	20	24920	3	*ANKMY1*, *DUSP28*, *RNPEPL1*	Very rare	31	7	23	Partial gene dup for *ANKMY1* and *RNPEPL1*, gene dup for *DUSP28*	**0.0007**	0.3229	
3p21.2	51989546	51995419	11	10143	3	*GPR62*, *PCBP4*	Amish specific	72	17	47	Partial gene dup	**0.0412**	**0.0238**	
3p25.3	11411823	11414339	5	2517	0&1	***ATG7***	Rare	46	13	24	Intronic del	0.7557	0.7020	Frontotemporal dementia, Parkinsons disease [45]
3q29	193136358	193140348	9	3991	0&1	***ATP13A4***	Rare	41	9	18	Intronic del	0.7389	0.2519	Autism [46]
4q22.1	91907363	91913329	16	10710	1	*FAM190A*	Amish Specific	34	6	21	Intronic del	**0.0099**	0.0794	
5q35.3	179221537	179238794	25	32170	3	***LTC4S***, *MGAT4B*, *MIR1229*, ***SQSTM1***, *MAML1*	Amish specific	79	17	36	Partial gene dup for *LTC4S* and *SQSTM1*, gene dup for *MGAT4B* and *MIR1229*	0.6641	0.0973	Venous thromboembolism and ischaemic stroke [47], Paget disease of bone [48]
5q35.3	179211629	179231681	25	32170	3	***LTC4S***, *MGAT4B*, *MIR1229*	Amish specific	61	14	36	Gene dup	0.2513	0.0973	Venous thromboembolism and ischaemic stroke [47]
6p21.32	32610719	32614917	10	4199	1	*HLA*-*DQA1*	Rare	25	7	13	Exonic del	1	0.0574	
6p25.3	1612234	1620037	15	9536	3	***FOXC1***	Very rare	47	14	23	Exonic dup	1	0.5815	Axenfeld-Rieger anomaly [49]
6q26	163041460	163139315	64	99376	1	***PARK2***	Very rare	21	5	11	Exonic del	0.5637	0.5534	Parkinsons disease [50], Autism [51]
7q22.1	100968058	101063059	159	183210	3	*EMID2*	Rare	100	25	38	Exonic dup	**0.0330**	0.7031	
7q36.1	149461487	149516968	67	55482	3	*SSPO*, *ZNF467*	Amish specific	154	39	68	Partial gene dup	0.8907	0.1100	
8p21.3	21943602	22024523	80	62524	3	*FAM160B2*, ***HR***, *NUDT18*	Amish specific	112	20	59	Partial gene dup for *FAM160B2* and *HR*, gene dup for *NUDT18*	**0.0284**	0.9422	Alopecia universalis [52], Congenital Atrichia [53]
8p22	15947559	16023673	118	76115	1	***MSR1***	Rare	19	3	7	Partial gene del	0.0833	0.6317	Prostate cancer [54]
8p22	15419777	15432653	24	21108	1	***TUSC3***	Very rare	19	3	7	Intronic del	0.0833	0.6317	Intellectual disability [55]
9q34.11	130497180	130518716	22	29513	3	*SH2D3C*, *TOR2A*	Amish specific	59	7	33	Partial gene dup for *TOR2A*, gene dup for *SH2D3C*	**0.0116**	0.4779	
10q11.21	45222200	45359483	125	151274	3	*TMEM72*-*AS1*	Rare	38	11	15	Partial gene dup	0.4386	0.5237	
10q21.3	68239474	68422442	209	182969	1	***CTNNA3***	Very rare	19	5	10	Partial gene del	0.6547	0.5957	Arrhythmogenic right ventricular cardiomyopathy [56]
11p11.2	45916436	45931646	24	29093	3	*C11orf94*, ***MAPK8IP1***, ***PEX16***	Amish specific	46	8	28	Partial gene dup for *MAPK8IP1* and *PEX16*, gene dup for *C11orf94*	**0.0197**	**0.0402**	Diabetes type 2 [57], Zellweger syndrome [58]
11p15.4	8959020	8964938	11	5919	1	*ASCL3*	Very rare	28	6	12	Gene del	0.4795	0.3681	
13q34	112712459	112726336	26	26199	3	*SOX1*	Amish specific	109	23	53	Gene dup	**0.0254**	0.0749	Neuronal development [59]
13q34	114518789	114530659	31	17552	3	*GAS6*	Very rare	92	21	45	Partial gene dup	0.6946	0.6193	
14q23.2	63957653	63962909	10	6398	1	*PPP2R5E*	Very rare	23	5	12	Intronic del	0.2568	0.1877	
15q11.2	24345146	24496990	76	152110	1	*PWRN2*	Rare	48	20	15	Gene del	0.1967	0.4189	Prader-Willi region [60]
15q26.1	90615898	90636762	28	26809	3	***IDH2***, *ZNF710*	Amish specific	27	8	13	Partial gene dup	0.7389	0.3569	D-2-hydroxyglutaric aciduria, type II [61]
16p12.1	27337036	27350687	15	20228	0&1	*IL4R*	Very rare	31	9	15	Exonic del	0.7812	0.1062	
17p13.3	811982	1183612	665	456481	3	*ABR*, *BHLHA9*, *MIR3183*, *NXN*, *TIMM22*, *TUSC5*	Very rare	23	3	12	Partial gene dup for *NXN* and *TUSC5*, gene dup for *ABR*, *BHLHA9*, *MIR3183* and *TIMM22*	0.6547	0.3319	
18q23	77150335	77162816	40	24952	3	***NFATC1***	Amish specific	41	13	23	Exonic dup	0.8273	0.1415	Tricuspid atresia [62]
18q23	76725624	76767375	35	41752	3	*SALL3*	Very rare	37	11	30	Gene dup	0.1336	0.0864	
18q23	77241092	77251061	21	16450	3	*NFATC1*	Amish specific	31	13	19	Exonic dup	0.3938	0.1313	
21q22.3	44822871	44868895	35	46025	3	*SIK1*	Rare	95	22	38	Gene dup	0.2800	0.5553	

CNVs shown are rare in controls (present in fewer than 5% of controls. Rare: <5%, Very rare: <1%, Amish Specific: not found in controls), and common in the Amish (present in more than 5% of individuals). Contained genes shows all genes in CNV, disease genes are highlighted in bold. FBAT and EMMAX p-values for association analysis for Bipolar disorder are included, p<0.05 are in bold.

Next, we focused on the analysis of CNVs encompassing known disease genes. It has been suggested that heterozygosity for several mutations in Mendelian disease genes may lead to complex disease risk, such as behavioral anomalies in neurodevelopmental and psychiatric disorders [63]. To ask if CNVs in disease genes may contribute to the allelic architecture in the Amish family segregating bipolar disorder, we mapped known disease loci with respect to CNV breakpoints. Specifically, we utilized the known disease causing variants (classed ‘DM’ in HGMD) from the Human Gene Mutation Database to define 3457 genes associated with disease. We identified 81 CNV regions that overlap with genes with known disease causing mutations (Additional file 1: Table S3). Of these, 27 CNV regions are specific to the Amish pedigree, and 40 are rare (<5%) in the control population. Interestingly, the number of CNV regions that encompass disease genes shows a trend towards an increased burden in bipolar narrow phenotype individuals (narrow phenotype burden: 5.7, unaffected burden 4.8, p = 0.06), and this is also true for rare (including Amish specific) CNVs encompassing disease genes (narrow phenotype burden: 5.0, unaffected burden 4.0, p = 0.06) (Table 2).

In particular, we explored the transmission (from parent to child) for rare CNV deletions in disease genes. Additional file 1: Table S4 details the 12 CNV deletions found in these genes, many of which have a behavioral disease phenotype (Parkinson disease, Autism Spectrum Disorder, Intellectual disability). Additional file 1: Figure S2 displays the extensive genetic heterogeneity within this founder pedigree, focusing on the CNVs in these 11 genes. Different branches of the pedigree carry different CNVs, with some nuclear families carrying up to 5 rare deletions in a known disease associated gene. In addition, we find a previously identified schizophrenia associated CNV (17q12del) [64] in an individual with a BPII phenotype. It is striking that in this individual this CNV maps in the vicinity of a recombination site on the paternal chromosome. These results together provide evidence for a complex role of CNVs in the phenotypic presentation within this family.

Within this set of disease genes, we found an Amish exclusive duplication in the *HOXD* cluster, present in 36 families. Of those with bipolar disorder in these carrier families 15/54 (28%) have the CNV, compared to around 24/144 (17%) of well individuals. Our analysis also detected three CNVs in genes previously linked to recessive disease in the Plain populations (*CNTNAP2*: [65]; *ADAMTS10*, *CLCNKB*: [32]). These include a rare intronic heterozygous duplication (present in two individuals, including one affected) in contactin associated protein-like 2 (*CNTNAP2*), a gene associated with autism spectrum disorder. In addition, four individuals (one affected) have an exonic heterozygous deletion not found in controls in ADAM metallopeptidase with thrombospondin type 1 motif (*ADAMTS10*), a candidate gene for Weill-Marchesani syndrome. Lastly, 12 individuals (four affected) were found to have an exonic heterozygous duplication of the chloride channel, voltage-sensitive Kb (*CLCNKB*), variants in which are associated with essential hypertension.

The availability of a combined dense genotype and whole genome sequence for 30 parent child trios [33], permitted the investigation of a combined effect of CNVs with likely deleterious SNPs on the same and in *trans* haplotype. Table 4 lists 26 disease genes with both a CNV and at least one non-synonymous SNP present in the same gene in the same individual. Among CNVs in known disease genes are two rare CNVs at the *PARK2* locus: a 63 kb deletion and a 99 kb deletion spanning the second exon. Although these CNVs do not segregate with a bipolar disease status, it is notable that 300 kb distal to the CNV breakpoint, in the neighboring PARK2 co-regulated gene (*PACRG*), we previously detected a cluster of SNPs with a family-based association signal of p-value 2.16x10-6 for the top SNP (rs9365506) (Additional file 1: Figure S3) [33]. In addition, there are six individuals who have both an exonic missense variant (rs1801582) and a CNV in *PARK2*. The variant is located upstream of the CNV and in each individual is present on a different haplotype from the CNV. Haplotype analysis of the region shows multiple haplotypes containing the SNPs from the family-based association analysis, two of which contain the CNVs, further supporting a proposed clustering of several potential risk alleles (SNPs and CNVs) in a defined chromosomal region [33].

**Table 4:** **Compound heterozygosity** - **Disease genes with CNVs and variants in the same individual**

**Gene**	**Number of affected** **(Total** = **69)**	**Number of unaffected** **(Total** = **203)**	**CNV frequency**
*ATG7*	13	26	Rare
*SNTG1*	4	2	Common
*PTPRD*	4	3	Rare
*IL4R*	9	20	Rare
*HLA*-*DQA1*	7	15	Rare
*KCNJ6*	1	0	Amish Specific
*CCDC50*	6	15	Common
*DICER1*	1	1	Amish Specific
*GALNTL4*	1	1	Rare
*ATP13A4*	9	25	Rare
*ERBB4*	1	3	Common
*MSR1*	1	3	Rare
*PARK2*	1	3	Rare
*RHD*	2	6	Common
*CDH13*	0	1	Rare
*PRKG1*	0	1	Rare
*WWOX*	3	10	Common
*SMARCA2*	2	8	Common
*CYP2D6*	0	3	Rare
*TUSC3*	3	14	Rare
*UGT1A7*	0	7	Rare
*UGT1A8*	0	7	Rare
*UGT1A10*	0	8	Rare
*UGT1A3*	0	8	Rare
*CTNNA3*	0	9	Rare
*CACNA1C*	0	14	Common

Counts in each column represent the number of individuals with both a CNV and another variant in the same gene.

Also among these variants was a rare 2 kb intronic deletion in autophagy related 7 (*ATG7*), which is present in 46 individuals, out of which 13 have a bipolar phenotype (13/36, 36.1%; unaffected 25/124, 20.2%). Furthermore, individuals with this CNV also have a possibly damaging exonic missense SNP (rs36117895) present on the same haplotype as the CNV. We also identified a number of individuals (n = 19, affected 3/6, 50%; unaffected 14/39, 35.9%) with a rare CNV in the intron of tumor suppressor candidate 3 (*TUSC3*). These individuals carry a SNP (rs1035972) within the same gene on the same haplotype as the CNV. Although many of these individuals are ‘unaffected’ (14 unaffected, 3 affected) within our pedigree, mutations such as these may contribute to the overall burden of disease within the Amish population.

## Discussion

We recently reported a combined analysis of dense genotypes and whole genome sequence for a large Old Order Amish pedigree with bipolar disorder. This study focused on the analysis of missense mutations within linkage peaks and detected a high degree of genetic heterogeneity of mental illness in this family [33]. Here we report results of the analysis of CNVs in the same extended pedigree. The mean burden per individual and size of CNVs were similar between our Amish sample and the European controls. While previous studies of the role of CNVs as risk alleles for bipolar disorder have been limited, an increased burden of CNVs in bipolar disorder has been reported by some [26,27], although these findings are not consistent [28,29]. We find a trend towards an increased burden of CNVs in genes in individuals with BP, specifically for CNVs that are rare or moderately frequent in the general population. While this finding does not reach significance, it adds to a body of evidence that suggests that CNVs may have some as-yet undefined role in BP and should be investigated accordingly.

We identified 13 CNVs in genes previously associated with psychiatric and developmental disorders, that are present at a higher rate in the Amish extended pedigree than in the control sample. The comparison of the frequency of these structural anomalies in the extended pedigree and in a large control dataset identified disease associated CNVs that are enriched in this population and may serve as a starting point for a future “Genotype-first approach” [66] to defining subtypes of bipolar and other complex diseases in this founder population. For example, rare CNVs were found in *SOX1*, a transcriptional activator thought to play a role in neuronal development [59]; and near *GM2A*, which is highly expressed in the brain and can harbor mutations which result in a variant of Tay-Sachs disease [67]; and *SLC6A15*, an amino acid transporter expressed highly in the brain and associated with major depressive disorder [68]. The largest rare, genic deletion in our pedigree, encompassing *PWRN2* and the surrounding region, was found more frequently in bipolar individuals within carrier families. *PWRN2* lies within a 1.5 Mb section on the long arm of chromosome 15 found to be deleted in Prader-Willi syndrome, a neurogenetic disorder with cognitive, behavioral and endocrine phenotypes [69]. A duplication in the *HOXD* region on chromosome 2 was also present more frequently in bipolar individuals. The *HOXD* genes play important roles in morphogenesis, and deletions in this cluster have been associated with limb and genital abnormalities [70]. Our study design allowed us to interrogate a combination of CNVs and other inherited mutations found within the same gene in a single individual on the same or opposite chromosome. Using this method, we identify 26 known disease genes that contain both a CNV and exonic missense SNP in one or more individuals. Of these, particular genes of interest include *ATG7*, which has been associated with frontotemporal dementia [71]; *TUSC3*, a gene associated with intellectual disability [50]; and *PARK2*, a gene associated with Parkinson’s disease [55,72]. Although we do not provide evidence that these CNVs and SNPs alone are disease causing in this pedigree, they may contribute together with other variants within the same chromosomal region to the disease risk [33]. In addition, molecular studies of these reported CNVs would be needed to determine if they have any effect on the gene. Larger studies in a non-Amish population are also required to determine if CNVs at these loci could be of relevance to bipolar or psychiatric disorder in a general population.

Although clinical information for the large extended Old Order Amish pedigree is limited to mood disorders, our genetic data permits the analysis of CNVs in genes associated with Mendelian diseases. In our initial report on the analysis of CNVs in the core Amish pedigree (in 50 family members), we provided proof of principle for a family-based investigation of a combination of structural variants in the same subject as that could confer risk for a disease [35]. Our reported trend for an increased burden of disease CNVs in bipolar family members (when compared to their unaffected relatives) needs to be further investigated with a larger sample size, both in the founder and general population. In many Mendelian diseases, psychiatric and behavioral symptoms are prevalent [73] and a wide range of medical co-morbidities are common in psychiatric disorders [63]. The variants underlying Mendelian disease are generally highly penetrant and less influenced by the environment, while haploinsufficiency or heterozygosity at *several* Mendelian disease loci may lead to complex behavioral anomalies in psychiatric disorders. Moreover, such a burden of risk alleles could explain the high degree of heritability but rather complex genetic architecture observed in these disorders. In other words, we propose that some of the *hidden heritability* may reside in gene-by-gene interactions and that the analysis of interactions at *bona fide* disease genes may be well powered by focusing on the impact of genetic variation within these critical classes of genes.

We have identified over 100 CNVs with a significant difference in allele frequency between the Amish family and the control sample. These structural variants add to an extended list of non-synonymous, likely deleterious variants that are rare in the 1000 Genomes project dataset (<2%), but present in 1-30% of BP subjects and their family members [33]. Owing to the anonymized fashion in which our study was conducted, it will not be possible, at this time, to evaluate possible phenotypic consequences of private Amish structural variants, those that are present in >10% of family members in this pedigree, but rare or absent in the general population. However, several ongoing genetics research initiatives involving the Plain populations [33,34] combined with the clinical genetics profiling applied in several clinics that serve these communities, are generating valuable insights that could potentially allow prevention of disability and disease. As reported by the Clinic For Special Children and by colleagues involved in genetic studies in Hutterites, providing education and offering clinical carrier status for devastating Mendelian diseases would likely be welcomed by members of the founder communities [74,75].

Our study has multiple limitations. First of all, the analysis is focused on a large extended family and power is limited for the statistical assessment. Also, it is difficult to determine if CNVs found to be enriched in the pedigree are unique to this founder population or to a cluster of these families, originating primarily from the Lancaster area (in Pennsylvania). However, the use of a genetic isolate and a large family structure provided us with a higher level of genetic and phenotypic homogeneity, and permitted the tracing of CNV events within nuclear families and across generations. Also, although the availability of biomaterials through a public cell repository represents a major advantage of this collection, DNA isolated from lymphoblastoid cell lines rather than blood represents a limitation of our study. Therefore, to avoid possible cell line artifacts, we excluded all singleton CNVs from our analysis and we were not able to address the role of *de novo* CNVs. Other studies have reported a role for *de novo* CNVs in BP [27], and we were unable to address this area of research. We note that we utilized CNVs from an eye disease study (AREDS) as a comparison for our dataset. Although we were not directly comparing the levels of CNVs between datasets, a disease free control dataset would have been more desirable. However, as their primary use was to identify CNVs that are present in the Amish more frequently than a European population, we believe the AREDS dataset was adequate for this purpose. Finally, the CNVs reported here were not experimentally validated, but due to our ability to show inheritance of the CNV from parent to child through the pedigree, we consider them to be validated CNVs [76].

## Conclusions

In summary, we identify a number of CNVs in an Old Order Amish pedigree segregating BP. Many of the CNVs found were rare in the general population and present in a large number of Amish individuals. Some of the CNVs were found in a higher frequency in individuals with a BP phenotype, and within genes known to play a role in human development and disease. We conclude that these CNVs may be contributing factors in the phenotypic presentation and heterogeneity of mental illness in this family.

## Methods

### Sample

The genetic-epidemiologic study of bipolar disorder among the Old Order Amish in Pennsylvania (The Amish Study of Major Affective Disorder) has been well documented [77,78]. Diagnostic methods included structured interviews (SADS-L) that were conducted with the patients and close others. In addition, medical records were obtained following signed, informed consent, these were abstracted and collated for five members of the Psychiatric Board who were blind to patient names, pedigree, address, admission/discharge diagnosis and treatment. The Psychiatric Board members used strict Research Diagnostic Criteria (RDC) and the Diagnostic and Statistical Manual of Mental Disorders, 4^th^ Edition (DSM-IV) for uniform clinical criteria, and reviewed all material every few years as a reliability check on diagnoses. The majority of affected individuals in the current pedigree are diagnosed as either BPI, BPII, or Major Depressive Disorder (MDD, recurrent) with a few Schizoaffective Disorder, BP Subtype, although there is a wider spectrum of Major Affective Disorders in the extended Amish pedigrees. In this study we place individuals into a number of phenotype categories for analysis: Narrow (BPI and BPII only), Broad (BPI, BPII and MDDR), and well (unaffected only).

Collection of blood samples followed diagnostic consensus. Lymphoblastoid cell lines were established by the Coriell Institute of Medical Research (CIMR). Signed informed consents were obtained to access medical records for the Amish Study clinicians exclusively to do diagnostic evaluations and clinical studies. Two forms were used: a) one with yearly Institutional Review Board (IRB, University of Miami) approval adhering to special guidelines because the Amish are defined as a “vulnerable” population; and b) a second using state approved, medical record consent forms for specific mental health clinics and psychiatric hospitals throughout central Pennsylvania. Collection of blood/tissue samples followed diagnostic consensus, using two informed consent forms: a) one with annual Univ. Miami IRB approval defining (with language appropriate for Old Order Amish) how their cells would be preserved for medical research on Major Affective Disorders; and, b) the Informed Consent Form required by the Institute for Medical Research (CIMR), later Coriell - National Institute for General Medical Sciences (NIGMS) Human Genetic Cell Repository (HGCR). In addition, analysis of whole-genome sequence data from consented individuals in this pedigree was also approved by the IRB of the Weill Cornell Medical College and the Perelman School of Medicine at the University of Pennsylvania.

### Control subjects

Control subjects were selected from the Age-Related Eye Disease Study (AREDS) sponsored by the National Institutes of Health (National Eye Institute). This prospective study of about 3600 participants follows the clinical course of age-related macular degeneration (AMD) and age-related cataract. Participants in this study were required to be ‘free of any illness or condition that would make long-term follow-up or compliance with study medications unlikely or difficult’ and as such are considered ‘well’ for the purposes of our study of mental illness. In addition, age of participants recruited to this study was between 55 to 80 years old, beyond the age at which presentation of a bipolar phenotype is to be expected. The individuals studied here were not affected with macular degeneration or cataract at the AREDS baseline examination.

Collection of blood samples for genetic research was performed following recruitment. Lymphoblastoid cell lines were established by the Coriell Institute of Medical Research (CIMR). Genotyping was performed on 2159 AREDS samples using Illumina Omni 2.5 M SNP arrays at the Center for Inherited Disease Research (CIDR). We performed rigorous quality control of the raw genotype calls by applying a series of filters on both markers and samples using PLINK [79]. The initial dataset contained 2,443,179 SNPs and 2159 samples. The following filters were applied in sequence; the numbers of markers or samples excluded is given in parentheses: a) exclude SNPs with missing rate > 0.5 (34,066), b) exclude samples with missing rate > 0.02 (0), c) exclude SNPs with missing rate > 0.02 (41,053), d) exclude SNPs with MAF < 0.02 (863,429), and e) exclude SNPs deviating from Hardy-Weinberg equilibrium at p < 1-e6 (34,405). After quality controls we retained 1,470,226 SNPs and 2159 samples. We subsequently performed Multi Dimensional Scaling on a set of ~ 500 k overlapping SNPs for all available AREDS genotypes and 1000 Genomes data. This analysis permitted the selection of 1897 subjects with European ancestry (Additional file 1: Figure S1) for further CNV calling and analysis.

### Genotyping

Genotyping was performed on 394 samples from the extended Amish pedigree using Illumina Omni 2.5 M SNP arrays at the Center for Applied Genomics (Children’s Hospital of Pennsylvania, Philadelphia, PA). As with the AREDS data, we performed rigorous quality control of the raw genotype calls by applying a series of filters on both markers and samples using PLINK [79], with the addition of excluding markers based on informative missingness and individuals/markers based on the mendel error rate. The initial dataset contained 2,379,855 SNPs and 394 samples. The following filters were applied in sequence; the numbers of markers or samples excluded is given in parentheses: a) exclude SNPs with missing rate > 0.5 (19,435), b) exclude samples with missing rate > 0.02 (6), c) exclude SNPs with missing rate > 0.02 (31,678), d) exclude SNPs with MAF < 0.02 (1,018,805), e) exclude SNPs with informative missingness p < 1e-6 (0), f) exclude SNPs deviating from Hardy-Weinberg equilibrium at p < 1-e6 (0), g) exclude individuals with >5% Mendelian errors (0) and h) exclude SNPs with >1% Mendelian errors (1334). After quality controls we retained 1,309,937 SNPs and 388 samples.

Association analysis for all CNV regions was performed using two different methods: a) FBAT [40] (Version 2.0.4), a version of the transmission distortion test adapted for larger families and b) EMMAX [41] (Version from February 2012), a statistical test for association analysis using mixed models that accounts for the population structure within the sample.

### Identification of copy number variants (CNVs)

CNVs were called by PennCNV, a previously described CNV detection algorithm [36], using the GC model wave adjustment [80]. CNVs were removed if they had a value > 0.30 standard deviation of LRR (LRRSD), a waviness factor (WF) value > 0.05, or < 5 SNPs. Regions that are known to be highly unreliable for CNV calls, such as immunoglobulin regions and the centromeres/telomeres of chromosomes were excluded from the analysis (see Additional file 1: Table S1). Samples that had a total CNV number greater than 3 SD from the mean, or samples that showed evidence of aneuploidy, were also excluded. After quality control we retained a set of 18,986 CNVs in 375 individuals from the Amish sample, and 77,205 in 1,897 individuals from the AREDS sample.

### Inherited CNV regions

From all available Amish samples with genotype data, we selected 328 individuals that belong to a nuclear family (parent plus children, 54 parents and 274 children) to ascertain regions containing inherited CNVs. This method consists of two stages. First, we establish the CNV region boundaries from the CNV that has the greatest overlap with other CNVs in the same genomic region. All CNVs that overlap 50% with this CNV are considered part of that CNV region. Second, we trace the inheritance of a CNV region using the pedigree information. For a CNV region to be inherited, it must be present in both the child and at least one parent.

### Human disease catalog

The Human Genome Mutation Database (HGMD) catalogs known disease associated variants (http://www.hgmd.org/). Most of the clinical phenotypes in the database are monogenic diseases. In its most recent release (June 2013) it contains 141,000 different variants in ~5,700 genes (“HGMD disease genes”) [81]. We cataloged all CNV regions (detected by the analysis of dense genotypes for the 328 Amish family members) that partially or fully overlap 3457 HGMD disease genes (‘DM’ tag in HGMD).

### Whole genome sequencing

Whole genome sequencing (WGS) for 80 Old Order Amish family members (including 30 parent child trios) was performed by Complete Genomics Inc. (CGI; Mountain View, CA) using a sequence-by-ligation method [82]. Paired-end reads of length 70 bp (35 bp at each end) were mapped to the National Center for Biotechnology Information (NCBI) human reference genome (build 37.2) using a Bayesian mapping pipeline [83]. Variant calls were performed by CGI using version 2.0.3.1 of their pipeline. False discovery rate estimates for SNP calls of the CGI platform are 0.2–0.6% [82]. Gene annotations were based on the NCBI build 37.2 seq_gene file contained in a NCBI annotation build. The variant calls within the WGS were processed using the cgatools software (version 1.5.0, build 31) made available by CGI. The listvar tool was used to generate a master list of the 11.1 M variants present in the 80 Amish samples. The testvar tool was used to determine presence and absence of each variant within the 80 Amish WGS. Only variants with high variant call scores (“VQHIGH” tag in the data files) were included. For further QC measures see [33].

As described in Georgi et al. [33], we performed phasing and imputation of variants identified by WGS into the Omni 2.5 M SNP genotypes using the Genotype Imputation Given Inheritance (GIGI) software version 1.02. GIGI performs imputation of dense genotypes in large pedigrees based on a sparse panel of framework markers using a Markov Chain Monte Carlo approach. Overall performance of our imputation is comparable to the published report [84]. For a threshold on the genotype imputation posterior probability of 0.85, we observed overall concordance of ~0.96 with a call rate of ~0.50 As expected, imputation performance increases for sub-pedigrees with a higher number of samples with WGS, i.e. when considering only nuclear families with WGS samples the performance improves to concordance ~0.99 and call rate ~0.87 [33].

## Additional file

Additional file 1:
**Figures and Tables illustrating quality control measures, and supplementary results including additional CNV regions found in disease genes and on Chromosome X.**
