# Supplementary material for: Copy number variants encompassing Mendelian disease genes in a large multigenerational family segregating bipolar disorder
Source: BMC Genet. 2015 Mar 15;16:27. doi: 10.1186/s12863-015-0184-1 (PMC4382929; doi:10.1186/s12863-015-0184-1)
Supplement: Additional file 1: — Figures and Tables illustrating quality control measures, and supplementary results including additional CNV regions found in disease genes and on Chromosome X. [file 12863_2015_184_MOESM1_ESM.docx]

**Supplementary Information**

**Copy number variants encompassing Mendelian disease genes in a large multigenerational family segregating Bipolar Disorder**

**Rachel L. Kember^1^, Benjamin Georgi^1^, Joan E. Bailey-Wilson^7^, Dwight Stambolian^2^, Steven M. Paul^4,5, 6^ and Maja Bućan^1, 3^**

**^1^**Departments of Genetics, **^2^**Ophthalmology, **^3^**Psychiatry, Perelman School of Medicine, University of Pennsylvania, Philadelphia, PA; ^4^Appel Alzheimer's Disease Research Institute, Mind and Brain Institute, Weill Cornell Medical College, New York, NY; ^5^Computational and Statistical Genomics Branch, National Human Genome Research Institute, National Institutes of Health, Baltimore, MD.

*Corresponding author:

Maja Bucan, Ph.D.

Departments of Genetics and Psychiatry

University of Pennsylvania

Philadelphia, PA 19104

Phone: (215) 898 0020
E-mail: [bucan@upenn.edu](mailto:bucan@upenn.edu)

Supplemental Figure 1: MDS plot comparing the Old Order Amish sample, Control sample, and 1000G data. The Amish are most similar to the Controls and Europeans (see inset).


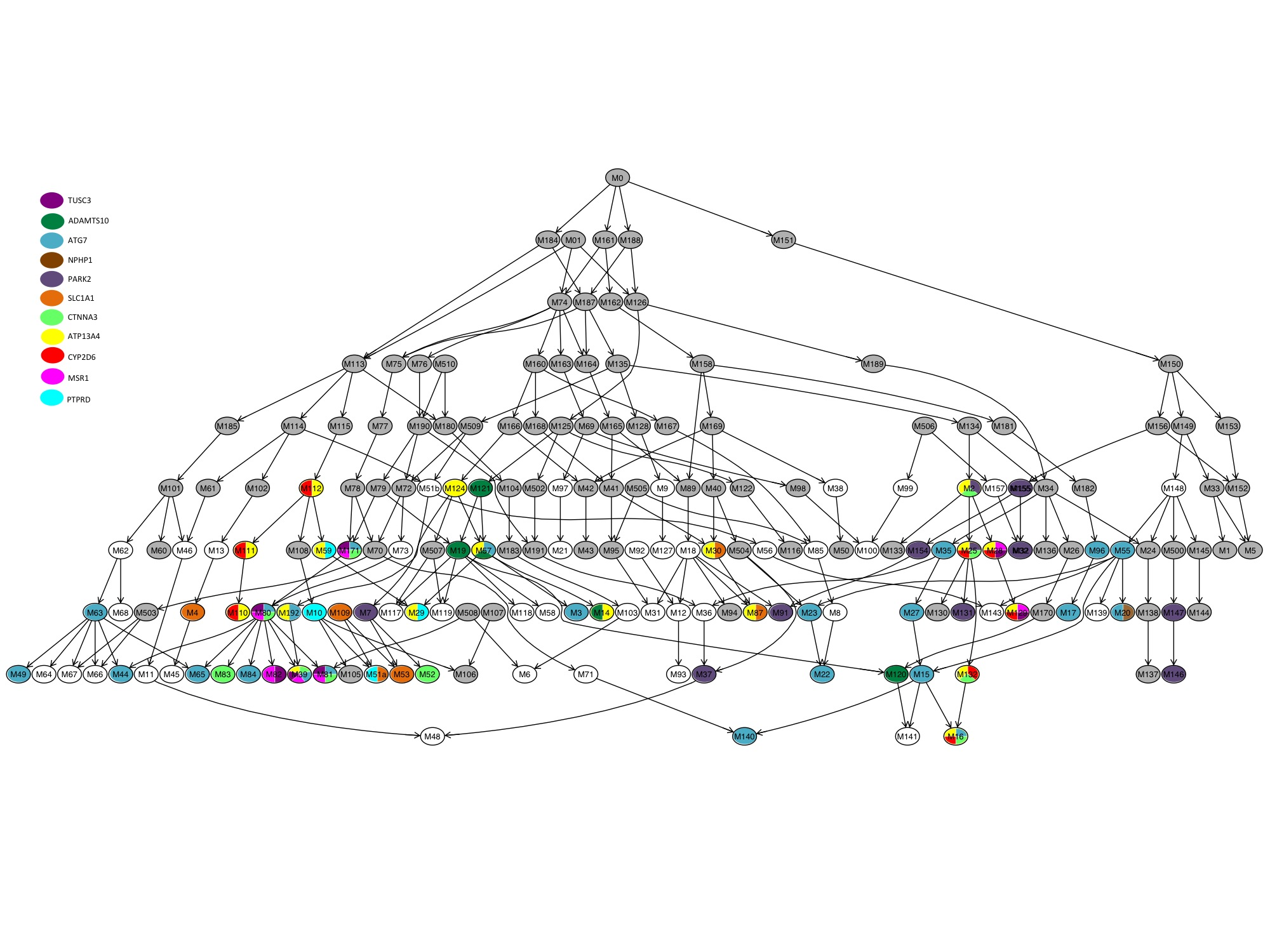


Supplemental Figure 2: Nuclear Family Graph depicting families that are carriers of rare deletions in disease associated genes. Each node represents a nuclear family and families are connected by parent-child relationships. Nuclear families without CNV data are colored in grey. Nuclear families with CNV data are either white (no rare deletions in disease associated genes) or multicolored (carry a rare deletion in one or many disease associated gene/s).


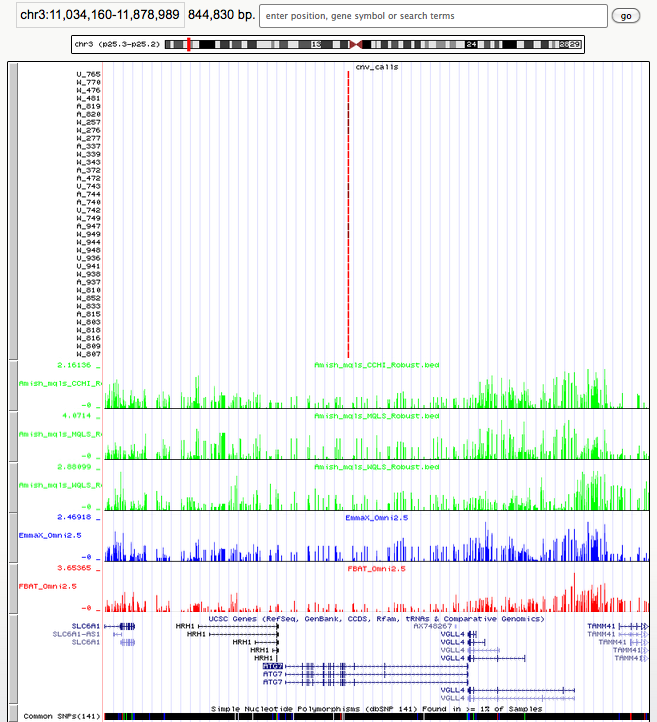

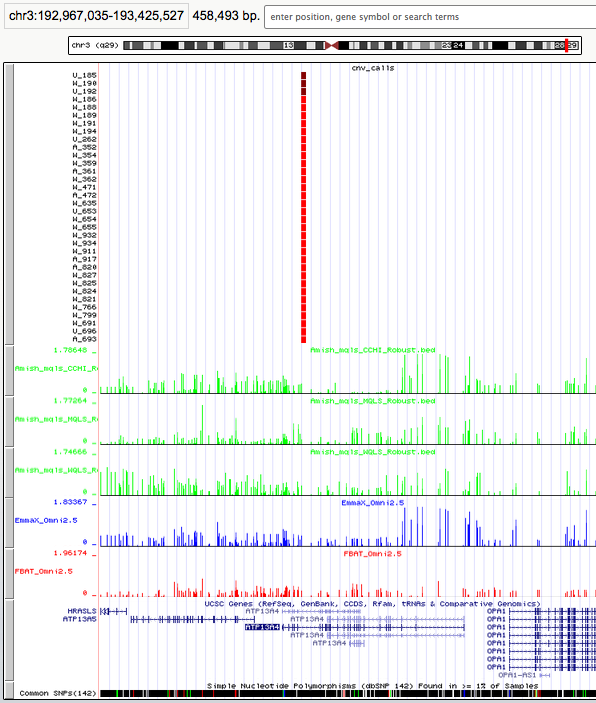

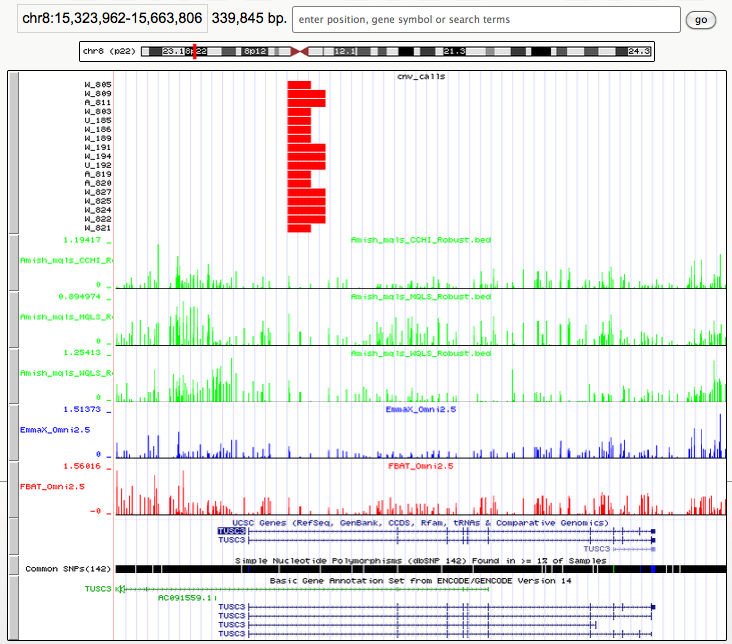

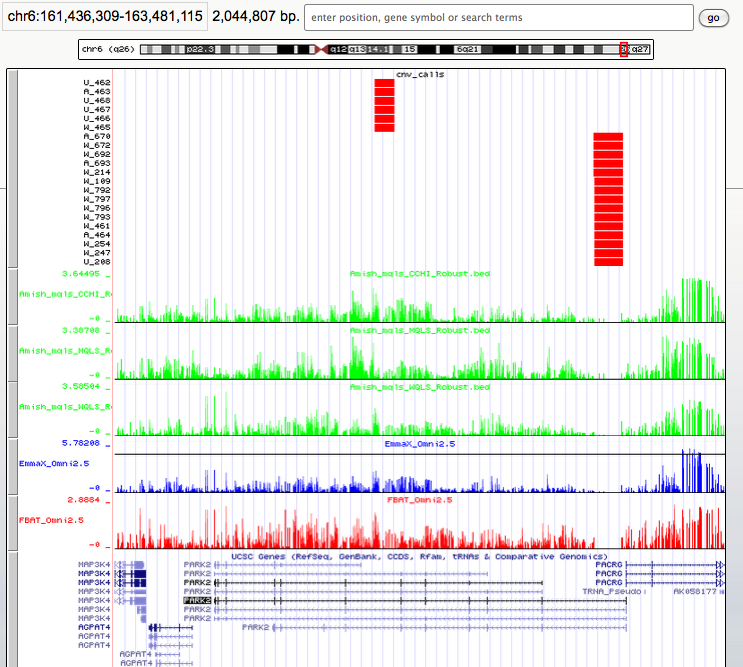


Supplemental Figure 3: UCSC Genome browser (<http://genome.ucsc.edu/index.html>) images of key CNV deletions: *ATG7, ATP13A4, PARK2, TUSC3, PWRN2*. From top: location of CNV deletion (in red), -log10 p-values for SNPs: MQLS (in green), EMMAX (in blue), FBAT (in red).


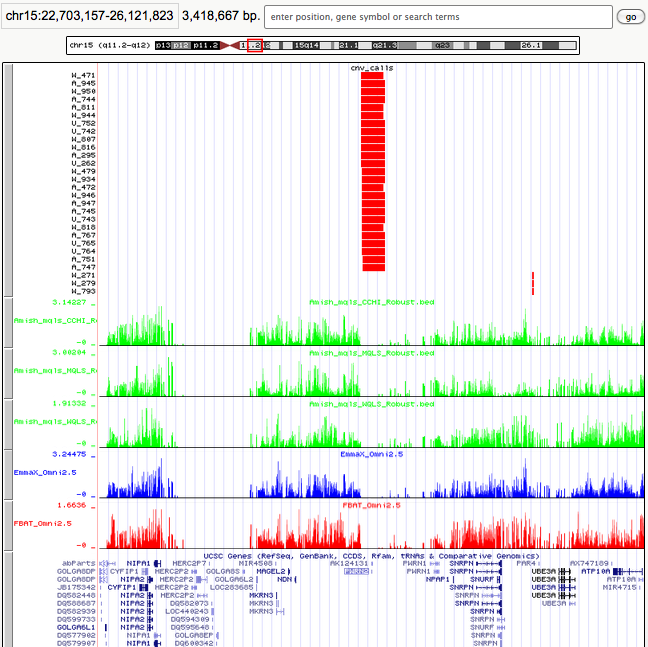


| Chromosome | Telomere | Centromere | Telomere | Immunoglobulin region |
| --- | --- | --- | --- | --- |
| chr1 | 1-500000 | 248750621-249250621 | 121181400-129160550 | - |
| chr2 | 1-500000 | 242699373-243199373 | 90232920-97164610 | 89156874-89630187 |
| chr3 | 1-500000 | 87627950-94467750 | 197522430-198022430 | - |
| chr4 | 1-500000 | 47849780-52994050 | 190654276-191154276 | - |
| chr5 | 1-500000 | 45673650-51144580 | 180415260-180915260 | - |
| chr6 | 1-500000 | 58373370-63807987 | 170615067-171115067 | - |
| chr7 | 1-500000 | 57577700-62027866 | 158638663-159138663 | - |
| chr8 | 1-500000 | 42734900-48494700 | 145864022-146364022 | - |
| chr9 | 1-500000 | 46953800-51015370 | 140713431-141213431 | - |
| chr10 | 1-500000 | 37661460-42723800 | 135034747-135534747 | - |
| chr11 | 1-500000 | 51173650-56055240 | 134506516-135006516 | - |
| chr12 | 1-500000 | 32932240-38602920 | 133351895-133851895 | - |
| chr13 | 1-500000 | 15777340-19848540 | 114669878-115169878 | - |
| chr14 | 1-500000 | 15694560-19506780 | 106849540-107349540 | 105994256-107281230,  22090057-23021097 |
| chr15 | 1-500000 | 15437000-21135250 | 102031392-102531392 | - |
| chr16 | 1-500000 | 34213950-38988990 | 89854753-90354753 | - |
| chr17 | 1-500000 | 21845280-26237630 | 80695210-81195210 | - |
| chr18 | 1-500000 | 14946180-19402170 | 77577248-78077248 | - |
| chr19 | 1-500000 | 23961580-29011510 | 58628983-59128983 | - |
| chr20 | 1-500000 | 25233990-29763880 | 62525520-63025520 | - |
| chr21 | 1-500000 | 10440580-14770320 | 47629895-48129895 | - |
| chr22 | 1-500000 | 11735240-18296700 | 50804566-51304566 | 22385572-23265082 |
| chrX | 1-500000 | 57813650-63417360 | 154770560-155270560 | - |

Supplemental table 1: Telomeric, centromeric, and immunoglobulin regions removed from CNV analysis

| CNV Region | # snp | length (bp) | CN | Type | # Subjects | # Affected | Contained genes |
| --- | --- | --- | --- | --- | --- | --- | --- |
| chrX:3199999-3216381 | 13 | 16383 | 1 | Very rare | 1 | 0 | *CXorf28* |
| chrX:16346245-16353797 | 9 | 7553 | 1 | Rare | 6 | 0 |  |
| chrX:41160469-41162216 | 5 | 1748 | 0 | Rare | 2 | 0 |  |
| chrX:48125009-48155807 | 10 | 30799 | 1 | Very rare | 7 | 0 | *SSX1* |
| chrX:75286248-75453328 | 56 | 188918 | 3 | Very rare | 7 | 0 | *CXorf26* |
| chrX:94387629-94427208 | 30 | 39580 | 1 | Very rare | 2 | 0 |  |
| chrX:104141996-104146341 | 6 | 4346 | 1 | Very rare | 4 | 0 | *IL1RAPL2* |
| chrX:140348507-140779598 | 80 | 431092 | 0 | Rare | 13 | 0 | *SPANXA1, SPANXA2, SPANXA2-OT1* |

Supplemental table 2: CNV regions on Chromosome X

| CNV Region | length (bp) | cn | Type | # Subjects | # Affected | Contained genes |
| --- | --- | --- | --- | --- | --- | --- |
| chr8:72215027-72217690 | 2663 | 0 | Common | 3 | 2 | ***EYA1*** |
| chr19:8660004-8672000 | 11996 | 1 | Amish specific | 4 | 1 | ***ADAMTS10*** |
| chr12:2245636-2259042 | 13406 | 1 | Common | 23 | 0 | ***CACNA1C*** |
| chr16:83197361-83209154 | 11793 | 1 | Rare | 9 | 1 | ***CDH13*** |
| chr10:68239474-68422442 | 182968 | 1 | Very rare | 20 | 5 | ***CTNNA3*** |
| chr14:95594207-95619101 | 24894 | 1 | Amish specific | 6 | 1 | ***DICER1****, MIR3173* |
| chr2:234648708-234658250 | 9542 | 1 | Very rare | 8 | 0 | *DNAJB3,* ***UGT1A10, UGT1A3, UGT1A4, UGT1A5, UGT1A6, UGT1A7, UGT1A8, UGT1A9*** |
| chr2:213187034-213196099 | 9065 | 1 | Common | 7 | 1 | ***ERBB4*** |
| chr11:11590291-11594572 | 4281 | 1 | Very rare | 4 | 2 | ***GALNTL4*** |
| chr6:32610719-32614917 | 4198 | 1 | Rare | 30 | 7 | ***HLA-DQA1*** |
| chr6:32458168-32527079 | 68911 | 1 | Common | 4 | 0 | ***HLA-DRB5****, HLA-DRB6* |
| chr21:39109477-39118267 | 8790 | 1 | Amish specific | 5 | 1 | ***KCNJ6*** |
| chr2:110850583-110982530 | 131947 | 1 | Very rare | 7 | 2 | *LINC00116, MALL,* ***NPHP1*** |
| chr8:15947559-16023673 | 76114 | 1 | Rare | 19 | 3 | ***MSR1*** |
| chr6:162308434-162372024 | 63590 | 1 | Very rare | 7 | 2 | ***PARK2*** |
| chr6:163041460-163139315 | 97855 | 1 | Very rare | 21 | 5 | ***PARK2*** |
| chr10:53204654-53211993 | 7339 | 1 | Rare | 4 | 0 | ***PRKG1*** |
| chr9:10523111-10527999 | 4888 | 1 | Very rare | 9 | 4 | ***PTPRD*** |
| chr1:25598276-25642596 | 44320 | 1 | Common | 33 | 5 | ***RHD*** |
| chr9:4566951-4574683 | 7732 | 1 | Very rare | 8 | 2 | ***SLC1A1*** |
| chr9:2148213-2153066 | 4853 | 1 | Common | 29 | 5 | ***SMARCA2*** |
| chr8:15419777-15432653 | 12876 | 1 | Very rare | 19 | 3 | ***TUSC3*** |
| chr3:11411823-11414339 | 2516 | 0,1 | Rare | 47 | 13 | ***ATG7*** |
| chr3:193136358-193140348 | 3990 | 0,1 | Rare | 41 | 9 | ***ATP13A4*** |
| chr3:191064647-191070300 | 5653 | 0,1 | Common | 26 | 6 | ***CCDC50*** |
| chr8:3786543-3790254 | 3711 | 0,1 | Common | 11 | 2 | ***CSMD1*** |
| chr16:27337036-27350687 | 13651 | 0,1 | Very rare | 32 | 9 | ***IL4R*** |
| chr7:142476094-142484042 | 7948 | 0,1 | Common | 28 | 9 | ***PRSS2****, PRSS3P2* |
| chr8:51031221-51038149 | 6928 | 0,1 | Common | 9 | 4 | ***SNTG1*** |
| chr22:42522638-42531210 | 8572 | 0,1,3 | Rare | 24 | 4 | ***CYP2D6*** |
| chr16:78372894-78383509 | 10615 | 0,3 | Common | 74 | 12 | ***WWOX*** |
| chr9:136319068-136344853 | 25785 | 3 | Very rare | 6 | 0 | ***ADAMTS13****, CACFD1, SLC2A6* |
| chr20:60871735-60892206 | 20471 | 3 | Amish specific | 31 | 8 | *ADRM1,* ***LAMA5*** |
| chr15:29036113-29981330 | 945217 | 3 | Very rare | 3 | 0 | ***APBA2****, FAM189A1, LOC646278, NDNL2* |
| chr15:29381175-29625939 | 244764 | 3 | Very rare | 3 | 0 | ***APBA2****, FAM189A1, NDNL2* |
| chr15:29036113-29264924 | 228811 | 3 | Very rare | 3 | 0 | ***APBA2****, LOC646278* |
| chr18:77137293-77162816 | 25523 | 3 | Amish specific | 16 | 6 | *ATP9B,* ***NFATC1*** |
| chr11:45916436-45931646 | 15210 | 3 | Amish specific | 52 | 10 | *C11orf94,* ***MAPK8IP1, PEX16*** |
| chr1:10685672-10714911 | 29239 | 3 | Amish specific | 29 | 8 | *CASZ1,* ***PEX14*** |
| chr17:77758484-77781725 | 23241 | 3 | Amish specific | 6 | 3 | ***CBX2****, CBX8* |
| chr16:55843252-55850708 | 7456 | 3 | Very rare | 14 | 3 | ***CES1*** |
| chr16:75539436-75578459 | 39023 | 3 | Rare | 9 | 2 | *CHST5,* ***TMEM231*** |
| chr1:16368481-16387466 | 18985 | 3 | Rare | 12 | 4 | ***CLCNKB****, FAM131C* |
| chr7:147520503-147620209 | 99706 | 3 | Very rare | 2 | 1 | ***CNTNAP2****, MIR548F3* |
| chr1:36561385-36562551 | 1166 | 3 | Amish specific | 4 | 0 | ***COL8A2*** |
| chr4:47735503-47917730 | 182227 | 3 | Amish specific | 8 | 2 | ***CORIN****, NFXL1* |
| chr19:18706504-18733355 | 26851 | 3 | Amish specific | 15 | 4 | ***CRLF1****, TMEM59L* |
| chr1:150683978-150704215 | 20237 | 3 | Amish specific | 18 | 4 | *CTSS,* ***HORMAD1*** |
| chr19:7948890-7994233 | 45343 | 3 | Very rare | 24 | 9 | *CTXN1, LOC100507588, LRRC8E, MAP2K7, SNAPC2,* ***TIMM44*** |
| chr22:24173141-24256334 | 83193 | 3 | Very rare | 9 | 3 | *DERL3, LOC284889, MIF, SLC2A11,* ***SMARCB1*** |
| chr22:18874965-19020529 | 145564 | 3 | Rare | 2 | 0 | *DGCR10, DGCR5, DGCR6, DGCR9,* ***PRODH*** |
| chr16:67232360-67241282 | 8922 | 3 | Amish specific | 18 | 7 | *E2F4, ELMO3,* ***LRRC29****, MIR328* |
| chr2:233345861-233358857 | 12996 | 3 | Amish specific | 7 | 1 | ***ECEL1*** |
| chr20:39985597-39994193 | 8596 | 3 | Very rare | 5 | 2 | *EMILIN3,* ***LPIN3*** |
| chr2:176929113-177000696 | 71583 | 3 | Amish specific | 50 | 15 | *EVX2, HOXD-AS2,* ***HOXD10****, HOXD11, HOXD12****, HOXD13****, HOXD8, HOXD9* |
| chr8:21943602-22024523 | 80921 | 3 | Amish specific | 115 | 21 | *FAM160B2,* ***HR****, NUDT18* |
| chr1:161570803-161619741 | 48938 | 3 | Common | 4 | 2 | ***FCGR3B****, HSPA7* |
| chr5:180043388-180079329 | 35941 | 3 | Amish specific | 31 | 7 | ***FLT4*** |
| chr14:38058924-38069828 | 10904 | 3 | Amish specific | 20 | 8 | ***FOXA1*** |
| chr6:1612234-1620037 | 7803 | 3 | Very rare | 49 | 15 | ***FOXC1*** |
| chr16:86600050-86615583 | 15533 | 3 | Amish specific | 3 | 0 | ***FOXC2****, FOXL1* |
| chr19:48198675-48206230 | 7555 | 3 | Amish specific | 10 | 3 | ***GLTSCR1*** |
| chr19:3082590-3126332 | 43742 | 3 | Very rare | 30 | 6 | ***GNA11*** |
| chr3:100345201-100441716 | 96515 | 3 | Rare | 2 | 0 | *GPR128,* ***TFG*** |
| chr9:127231503-127247931 | 16428 | 3 | Amish specific | 5 | 1 | *GPR144,* ***NR5A1*** |
| chr15:90615898-90636762 | 20864 | 3 | Amish specific | 29 | 8 | ***IDH2****, ZNF710* |
| chr17:44173505-44206665 | 33160 | 3 | Common | 7 | 1 | ***KANSL1*** |
| chr17:44213712-44347946 | 134234 | 3 | Rare | 23 | 10 | ***KANSL1****, KANSL1-AS1* |
| chr21:35769519-35906068 | 136549 | 3 | Very rare | 4 | 2 | ***KCNE1****, RCAN1* |
| chr19:33684895-33702245 | 17350 | 3 | Very rare | 20 | 3 | *LRP3,* ***SLC7A10*** |
| chr5:179211629-179231681 | 20052 | 3 | Amish specific | 65 | 15 | ***LTC4S****, MGAT4B, MIR1229, MAML1* |
| chr5:179221537-179238794 | 17257 | 3 | Amish specific | 83 | 18 | ***LTC4S****, MGAT4B, MIR1229,* ***SQSTM1****, MAML1* |
| chr19:18260710-18273410 | 12700 | 3 | Amish specific | 5 | 2 | *MAST3,* ***PIK3R2*** |
| chr1:155152205-155162067 | 9862 | 3 | Amish specific | 49 | 13 | ***MUC1****, TRIM46* |
| chr18:77150335-77251061 | 100726 | 3 | Amish specific | 80 | 29 | ***NFATC1*** |
| chr22:20214783-20235973 | 21190 | 3 | Very rare | 4 | 2 | ***RTN4R*** |
| chr16:87864621-87874736 | 10115 | 3 | Very rare | 26 | 8 | ***SLC7A5*** |
| chr1:246425984-246445421 | 19437 | 3 | Very rare | 16 | 4 | ***SMYD3*** |
| chr16:28614977-28620752 | 5775 | 3 | Common | 2 | 0 | ***SULT1A1*** |
| chr22:19738645-19763978 | 25333 | 3 | Very rare | 18 | 4 | ***TBX1*** |
| chr5:110434008-110444810 | 10802 | 3 | Very rare | 1 | 0 | ***WDR36*** |

Supplemental Table 3: CNV Regions in known disease loci. CNVs are categorized as Common (<5% in controls), Rare (>5% in controls), Very rare (>1% in controls) or Amish Specific (not found in controls). Disease genes are highlighted in bold.

| **CNV Region** | **Gene** | **# of families** | **Transmission to affected** | **Transmission to unaffected** | **Disease associated with gene** |
| --- | --- | --- | --- | --- | --- |
| chr2:110850583-110982530 | NPHP1 | 1 | 2 T from unaffected parent | 4 T from unaffected parent | Kidney diseases, Intellectual disability |
| chr3:11411823-11414339 | ATG7 | 12 | 2 T from affected parent  4 T from other phenotype parent  5 T from unaffected parent | 7 T from affected parent  2 T from other phenotype parent  12 T from unaffected parent | Parkinson Disease |
| chr3:193136358-193140348 | ATP13A4 | 11 | 1 T from affected parent  3 T from other phenotype parent  2 T from unaffected parent | 5 T from affected parent  7 T from other phenotype parent  11 T from unaffected parent | Autism spectrum disorder |
| chr6:162308434-162372024 | PARK2 | 1 | 1 T from affected parent | 1 T from affected parent | Autism spectrum disorder, Parkinson disease |
| chr6:163041460-163139315 | PARK2 | 6 | 2 T from affected parent  1 T from unaffected parent | 2 T from affected parent  1 T from other phenotype parent  8 T from unaffected parent | Autism spectrum disorder, Parkinson disease |
| chr8:15419777-15432653 | TUSC3 | 3 | 3 T from unaffected parent | 12 T from unaffected parent | Intellectual disability |
| chr8:15947559-16023673 | MSR1 | 3 | 3 T from unaffected parent | 12 T from unaffected parent | Oesophageal adenocarcinoma, Prostate cancer |
| chr9:4566951-4574683 | SLC1A1 | 1 | 1 T from affected parent | 6 T from affected parent | Dicarboxylic aminoaciduria, Psychosis |
| chr9:10523111-10527999 | PTPRD | 2 | 1 T from affected parent  2 T from other phenotype parent | 3 T from other phenotype parent | Ewing sarcoma |
| chr10:68239474-68422442 | CTNNA3 | 6 | 2 T from affected parent  2 T from unaffected parent | 2 T from affected parent  10 T from unaffected parent | Arrhythmogenic right ventricular cardiomyopathy |
| chr19:8660004-8672000 | ADAMTS10 | 2 | 1 T from unaffected parent | - | Weill-Marchesani syndrome |
| chr22:42522638-42531210 | CYP2D6 | 5 | 1 T from unaffected parent | 1 T from affected parent  10 T from unaffected parent | Cytochrome P450 deficiency, Schizophrenia |

Supplemental Table 4: Transmission of rare CNV deletions in disease associated gene
